# Comparing ownership and use of bed nets at two sites with differential malaria transmission in western Kenya

**DOI:** 10.1186/s12936-016-1262-1

**Published:** 2016-04-14

**Authors:** Kacey C. Ernst, Mary H. Hayden, Heather Olsen, Jamie L. Cavanaugh, Irene Ruberto, Maurice Agawo, Stephen Munga

**Affiliations:** Division of Epidemiology and Biostatistics, Mel and Enid Zuckerman School of Public Health, The University of Arizona, 1295 N. Martin Ave., Tucson, AZ 85724 USA; National Center for Atmospheric Research, 3450 Mitchell Lane, Boulder, CO 80301 USA; Centre for Global Health Research, Kenyan Medical Research Institute, Kisumu-Busia Highway, Kisumu, Kenya

**Keywords:** Malaria, Kenya, Bed nets, Use, Ownership, Challenges, LLINs, IRS

## Abstract

**Background:**

Challenges persist in ensuring access to and optimal use of long-lasting, insecticidal bed nets (LLINs). Factors associated with ownership and use may differ depending on the history of malaria and prevention control efforts in a specific region. Understanding how the cultural and social-environmental context of bed net use may differ between high- and low-risk regions is important when identifying solutions to improve uptake and appropriate use.

**Methods:**

Community forums and a household, cross-sectional survey were used to collect information on factors related to bed net ownership and use in western Kenya. Sites with disparate levels of transmission were selected, including an endemic lowland area, Miwani, and a highland epidemic-prone area, Kapkangani. Analysis of ownership was stratified by site. A combined site analysis was conducted to examine factors associated with use of all available bed nets. Logistic regression modelling was used to determine factors associated with ownership and use of owned bed nets.

**Results:**

Access to bed nets as the leading barrier to their use was identified in community forums and cross-sectional surveys. While disuse of available bed nets was discussed in the forums, it was a relatively rare occurrence in both sites. Factors associated with ownership varied by site. Education, perceived risk of malaria and knowledge of individuals who had died of malaria were associated with higher bed net ownership in the highlands, while in the lowlands individuals reporting it was easy to get a bed net were more likely to own one. A combined site analysis indicated that not using an available bed net was associated with the attitudes that taking malaria drugs is easier than using a bed net and that use of a bed net will not prevent malaria. In addition, individuals with an unused bed net in the household were more likely to indicate that bed nets are difficult to use, that purchased bed nets are better than freely distributed ones, and that bed nets should only be used during the rainy season.

**Conclusion:**

Variations in factors associated with ownership should be acknowledged when constructing messaging and distribution campaigns. Despite reports of bed nets being used for other purposes, those in the home were rarely unused in these communities. Disuse seemed to be related to beliefs that can be addressed through education programmes. As mass distributions continue to take place, additional research is needed to determine if factors associated with LLIN ownership and use change with increasing availability of LLIN.

## Background

Great strides have been made in the last decade to reduce the burden of malaria across sub-Saharan Africa [1–3]. While integrative malaria-control strategies are promoted to further reduce its incidence, adopting long-lasting, insecticide-treated bed nets (LLINs) is currently the primary strategy being used to control transmission [4]. LLIN distribution has replaced the historical distribution of untreated bed nets or insecticide-treated bed nets (ITNs) that required frequent retreating. Between 2008 and 2010, an estimated 289 million LLINs were distributed throughout sub-Saharan Africa [4].

Free mass distributions have been identified as the best means to reduce disparities in LLIN ownership, and subsequent campaigns have had a great impact on equalizing ownership across wealth classes [5–8]. However, in some regions, these disparities still remain [6, 9]. Mass distribution campaigns have been demonstrated to result in better equity in coverage than clinic-based or social marketing strategies [10]. Targeting of pregnant women and children under 5 years of age has also led to higher coverage in households that have one of these risk groups, and these groups have been the focus of a significant proportion of work on LLIN ownership [8, 11, 12]. As malaria strategic plans move towards universal coverage (optimal 1:1.6 ratios of LLINs to people [13]), understanding factors that prevent the general population from obtaining LLINs becomes increasingly important. Factors influencing risk are likely to be variable and dependent on the types of distribution programmes.

Ownership of an LLIN is clearly the first step, but also critical is the optimal use of the LLIN. While benefits of high LLIN coverage in the community extend beyond individuals sleeping under them, the highest protection is gained through regular nightly use of an LLIN [14]. Evidence is building about what happens to LLINs following mass distribution campaigns, however, there are mixed results from different studies. Some previous studies have indicated that use of distributed LLINs is low [5, 11, 15, 16] while others indicate that most LLINs that are distributed are used for their intended purpose [17–19]. Some evidence suggests that while there are many reported challenges in using LLINs, including shape, inconvenience, heat, and discomfort, most individuals report using their LLIN the night prior to the interview [17]. This however, varies by region. For example, in Burkina Faso, use declined over time following distribution and was related to schedule and inconvenience [20]. Investigations in different geographic areas are warranted to better understand differences in the factors driving differential uptake and use of LLINs.

In Kenya, malaria transmission is highly variable, and continues to be a serious threat to health. As a result, concerted efforts are being made to reduce the burden of the disease through widespread dissemination of LLINs and targeted indoor residual spraying (IRS). In 2003, IRS was recommended for administration in Kenya. This was followed shortly by the free distribution of ITNs/LLINs to pregnant women and children under 5 in 2006. In 2010 the policy shifted to universal distribution with the goal of one bed net for every two people in a household. Both clinic distribution as well as mass distributions have taken place throughout the nation though access has to LLINs has not been equal across the regions. Results of the 2008–2009 DHS survey indicate that LLINs demonstrate a range of ownership of at least one ITN in a household from 41.4 % in the former Rift Valley Province to 76.5 % in the former Nyanza Province. This is somewhat reflective of the differences in transmission in those areas. In 2010, highland areas in western Kenya which fall within what was formerly known as the Rift Valley Province, had a low-slide positivity rate of 3.3 % in children 3 months to 14 years old, while children of the same age range in lowland endemic areas of which Nyanza Province was a part had parasitaemia of 38 % [21]. An assessment of nationwide Demographic and Health Survey data indicated that approximately 12 % of children under the age of five slept without a bed net even when there was an unused bed net in the house [22].

In this paper findings from a study conducted in two areas in western Kenya are presented. One is located in a lowland endemic area and the other in a highland site with seasonal transmission. There were two primary objectives for the analysis; (1) to determine factors associated with household-level ownership of bed nets and (2) in households which reported owning a bed net factors associated with not using all available bed nets were determined.

## Methods

### Ethics statement

Institutional Review Board (IRB) approval was obtained from the University of Arizona and the National Ethics Review Committee/Kenyan Medical Research Institute (SSC Protocol No. 2491). Participants in community forums provided written consent for participation and were requested to keep the identities of participants and the information that they discussed confidential. No information was publicly reported that would identify any study participant. Written consent was obtained from the household head prior to interview for the cross-sectional survey.

### Study settings

This research took place in two sites: a lowland site, Kabar West and Kabar Central sub-locations in Kisumu County, Kenya, and a highland site in Nandi County which is comprised of three sub-locations; Tindinyo, Kiborgok and Chepsonoi. Sites were selected to represent two different transmission dynamics with the highland site representing seasonal transmission and the lowland site representing holoendemic transmission. The community forums took place in 2011, while the cross-sectional surveys took place in August 2012 shortly after the peak of malaria transmission in Nandi County.

Lowland holoendemic site (referred to as the “lowlands”: Kabar West and Kabar Central are located in Kisumu County within what was formerly known as the Nyanza Province. These sub-locations are located on the Kano Plain in western Kenya, 30 km east of Kisumu City. They are approximately 1200 m above sea level; malaria transmission in this area is holoendemic and occurs year-round. Residents are primarily of the Luo ethnic group and are subsistence farmers with some casual labour in nearby sugar cane plantations (Table 1). One community health center is located within the study site and is a distribution site for LLINs. Conversations with administrators indicate that they provided them primarily to pregnant women and children under five prior to our survey. In addition, the last mass distribution that took place before survey was conducted in June 2010.

**Table 1:** Description of study sites and variability in reported malaria transmission and environmental influences

Sub-location, Province	Estimated population size^a^	Estimated average yearly malaria incidence^b^	Altitude range	Household bed net ownership^a^ (%)	Malaria transmission pattern	Description of environment
Highland site: Tindinyo, Chepsonoi, and Kiborgok sub-locations, Kapkangani, Nandi North, Rift Valley Province	12,300	35 per 1000	1600–2100 m	37	Seasonal after short and long rains	Primarily rural, bisected by major highway with some commercial shops along roadside; permanent and semi-permanent streams; tea, maize
Lowland site: Kabar East and Kabar West, Nyanza Province	4300	400 per 1000	1100–1200 m	52	Holo-endemic – year round high levels of transmission with seasonal peak following the rains	Rural; agriculture (maize, rice, sugarcane); less animal husbandry than Kapkangani

^a^Based on study census from 2012

^b^Based on passive surveillance data from community clinics

Highland seasonal transmission site (referred to as the “highlands”: Tindinyo, Chepsonoi, and Kiborgok sub-locations are located approximately 15 km east of Kapsabet town in the western Kenyan highlands within the area formerly known as the Rift Valley Province. The altitude of this area ranges from approximately 1600–2100 m. Topography is variable in this region with hills and valleys. In adjacent areas this variation in topography has been critical in determining the distribution of malaria risk [23]. Malaria transmission here is low and unstable with acute seasonal peaks generally after the heavy rains in April and May. Kalenjin and Luhya are the primary ethnic groups in this region. The primary occupation is rural subsistence agriculture, while some residents work as casual labourers on local tea estates (Table 1). There is a large health center, Kapkangani Health Center which is located near the central part of the study area in the highland site. It is also a source of LLINs for pregnant women and children under 5. Due to shortages staff indicated that they were prioritizing women with children under age 1 year. Clinic staff were not aware of any large-scale mass distributions that had taken place within the three years prior to survey.

### Bed net terminology

While distribution of LLINs is currently the norm for malaria prevention, there still exist untreated bed nets and ITNs within these communities. The general term ‘bed nets’ is used throughout the paper to refer to community-owned bed nets, unless the discussion focused specifically on LLINs that were being distributed starting in 2010 in the lowland endemic study area. In referencing current literature on LLIN distribution programmes, the term LLIN is used.

### Community forum methodology

#### Sampling

Purposive sampling was conducted to ensure multiple viewpoints were represented in community forums. Participants were recruited from multiple venues. Village chiefs were notified of the community forums and were asked to disseminate this information to the community. The programme coordinators provided informative talks at the local *baraza* (village meetings), church organizations, women’s groups, men’s groups, and secondary schools. Interested individuals were asked to communicate directly with local field assistants or with the programme coordinator. All community forum discussion participants were required to have resided in the study sites for at least the previous calendar year to be eligible for inclusion. Community forums were held in each of the sub-locations (n = 5, two lowland and three highland) to ensure geographic coverage of the study sites.

A total of ten community forums were held, comprised of a total of 105 participants. Stratification by gender and age was initially considered but community health workers, clinic staff and community leaders indicated mixed gender community forums were appropriate and informal confirmation of this was provided by potential participants. Participants included roughly equivalent groups of men (n = 53) and women (n = 52). Both young adults (n = 55; 18–30 years old) and older adults (n = 50; >30 years old) were included in the forums (Table 2).

**Table 2:** Current challenges to using insecticide-treated bed nets as reported in community-forums

Key themes	Sub-themes	Focus groups reporting theme	Representative quote
Access	Distance	H2, H3, L10	“The health clinic is far from the community members, up to 7 km, therefore going to buy a net is far and it leads to impulse buying of other items needed.” H3
	Cost	H1, H2, H3, H5, L9	“Once distributed to the people on warranty no chances of getting replacement unless you dig deep into your pockets.” L9
	Age-restrictions	H1, H2, H3, H4, H5, H6, L10	“There are problems getting a bed net because they are only given at the health facility on the condition that they belong to pregnant women and children under 5 years” H5
	Stock-outs	H1, H3, L7	“The greatest problem is that statistics taken for bed net supply doesn’t correspond to the actual nets brought for distribution hence most people don’t get the bed nets.” L7
	Replacement	H1, H6	“In some region the bed nets are sold and others given freely.it therefore kill the morale of others to acquire the nets.” H6
Effectiveness	Wears out too soon	H1, H2, H4, H5, L7, L8, L9, L10	“After the washing of the nets the effectiveness of the mosquito repellant fade away and that make it not effective.” L7
	Difficult to maintain/damage easily	H1, H2, H3, H6	“Most nets get burned with tin lamps creating a hole making the net not effective to use” H3
	Quality of distributed nets	H2, L8, L9	“Some of the nets have less effective chemical repellent since they are not retreated and are [ineffective] even though not in bad condition” L9
Side effects	Suffocation	H2, L7, L8, L10	“It also suffocates small children and leads to difficulty in breathing.” L7
	Heat	H4, L8, L10	“We don’t always use a bed net because during the dry spell season it is hot and people do not use the bed net.” H4
	Rash/allergy	H1, H2, H3, H4, H5, H6, L7, L8, L9, L10	“Once one gets into contact with the net which the repellant is still strong, the skin rashes are developed causing discomfort to the skin.” L7
	Family planning	H2	“The belief that the chemical in the bed net is for family planning [keeps them from using it]” H2
	Bad dreams	H4	“Sleeping under a bed net gives one to dream bad dreams.” H4
Lack of knowledge	Don’t understand transmission or importance of using bed net	H1, H2, H4, H6	“[In our] culture [we] believe that we used to sleep without nets why use it now?” H2
	Not aware of how to maintain bed net	H1, H2, H3, H4, H6, L7, L8	“Those who distributed them never taught us how to use them.” L8
	Use for other purposes	H1, H2, H4, H5, H6	“A bed net is a multi-purpose item. Some use it for decoration and curtains, fishing, rope to tie animals.” H4
Logistical/convenience	Hanging	H1, H2, H3, H4, H5, H6, L7, L8, L9, L10	“Hanging a bed net is a problem especially one without a ring. It is difficult because at night when you are tired you simply sleep.” L10
	Not attractive	H1, H2	“Some people are attractive to certain colours when a bed net is dull they don’t go for it.” H1
	Difficult to keep clean	L8, L10	“They become dirty very easily and they are difficult to [re]hang” L10
	Sleeping space too small	H1, L8	“Squared bed net consume space therefore some rooms are not enough.” H1
	Believe only for use with bed	H3, H4	“Some believe that bed net is used only on bed net on sleeping on the floor.” H3

Key themes were identified a priori and sub-themes were determined during the analysis phase. *H/L#* refers to the community forum site (*H* highland, *L* lowland and the community-forum number)

### Community forum administration

Study team members facilitated the community forum discussions using an interview guide developed by study investigators. The flow of the discussion moved from general to more specific topics: general health concerns in the community, the relative importance of malaria transmission, perception of malaria risk, knowledge and use of control measures, use of bed nets (generally and specifically LLINs), and challenges to using bed nets. During the forums, probes were used by the moderator to encourage detailed conversations and seek clarification. Initial translations of the interview guide were reviewed by local community members to account for variability in dialects across the study areas. Community forums were recorded using a digital audio recorder.

### Qualitative data analysis

All community forums were transcribed and translated by trained local Kenyan collaborators who are fluent speakers of the language in which the data were collected. Three researchers (KE, MH, JC) independently read and listened to the transcriptions and worked with trained local study personnel to ensure accuracy of transcription and translation of materials and data immersion. The researchers then coded each community forum based on themes that were identified a priori using the socio-ecological model which examines how environmental and personal factors intersect to influence health and decision making [24]. A table of sub-themes was compiled delineating the challenges to effective bed net use. Then, each community forum was reviewed again and independently coded according to sub-themes arising in the questions.

### Cross-sectional survey methods

#### Sampling

Concurrent with an enumeration of the study population, a survey was administered to households within the study sites. Systematic sampling was conducted in the highland site, with every third house being recruited for participation in the survey following enumeration. No replacement was conducted if the household head refused. The study site in the lowland area comprised a much smaller study area, and all households were recruited for participation following enumeration.

#### Data collection

Surveys were orally administered by local field assistants in the language of preference indicated by the household head. General topics followed the health belief model [25]: knowledge of malaria and attitudes about malaria including risk perception and practices of prevention and control, cues to action, and barriers to action. Specific inquiries were made about bed net use and the perception of bed nets in the community. Details about the bed nets owned by the household were collected for up to three bed nets and included age of the bed net, where it was obtained, and if it was treated/untreated.

### Data preparation and statistical analysis

Duplicate households were removed from the dataset prior to analysis. A new variable was created to represent wealth of the households; this variable was produced by creating a wealth index using ownership of a radio, television, light, stove, car, bike, sofa set, and bed as indicators for wealth. Weights were provided for each of the durable goods in accordance with their relative monetary value. Monetary values were obtained from markets within or near the study sites. Results from the household totals were divided into quintiles across the sample. Frequencies of key bed net use indicators were determined by site in accordance with the World Health Organization. Percent differences in frequencies of potential explanatory factors and knowledge and attitudes towards malaria risk, and malaria prevention and control were determined between the two sites. Bed net ownership was defined as owning at least one bed net in the home regardless of the household size. Odds ratios, 95 % confidence intervals, and p-values were determined as the measure of association with ownership using multivariate regression models stratified by site. Potential influencing factors included: having a child under the age of five in the household, knowing someone who died of malaria, education level, level of wealth, and if malaria is considered a serious disease.

Bed net use was examined by comparing households that reported using all owned bed nets the night before to households which reported that at least one bed net was not used the night before and at least one household member had slept without a bed net the night prior. As only households with bed nets were included in this analysis the sample size was greatly reduced. In addition, not using an available bed net was a rare outcome and this limited the ability to stratify by study site and use multivariable models. Therefore, separate models were constructed for each hypothesized factor that could influence use of available bed nets. Each model was adjusted for potential confounding from several demographic variables including age, wealth and education levels. All of the statistical analyses were conducted using SAS 9.3.

## Results

### Community forum

#### Community forum characteristics

Community forum discussions were held in a range of sites, including schools (n = 4), at local health facility (n = 2), in town centre (n = 2), and local churches (n = 2). Community forums were conducted in both the highlands (n = 6) and lowlands (n = 4). Total attendance in the highlands was n = 65 and in the lowlands, n = 40. Community forums averaged 60 min. The average number of participants in each group was ten. Participants included roughly equivalent groups of men (n = 53) and women (n = 52). Both young adults (n = 55; 18–30 years old) and older adults (n = 50; >30 years old) were included in discussion groups. Themes identified a priori included access issues, side-effects, effectiveness, lack of knowledge, logistical difficulties (Table 2).

### Access issues

Ownership challenges were reported among participants in all of the highland community forums and three of the four lowland community forums. Sub-themes included distance (number of highland groups reporting sub-theme (H) = 2, and lowland groups reporting sub-theme (L) = 1), cost (H = 6, L = 1), age-restrictions (H = 6, L = 1), and stock-outs (H = 2, L = 1). People repeatedly noted that typically LLINs are only distributed to pregnant women, leaving many family members without a bed net. As one highland participant noted:

*“There are some problems getting a bed net because they are only given at the health facility on the condition that they belong to pregnant women and children under five years”.*

Mass distributions were not common and community members indicated that only one LLIN was received per household. Competing expenses made it difficult for people to afford to buy a LLIN commercially. One lowland participant noted that:

“*Due to economic pressure people consider buying food stuff rather than buying a bed net”.*

The distance from a health clinic also made it difficult for some highland participants:

*“The health clinic is far from the community members, up to 7* *km, therefore going to buy a net is far and it leads to impulse buying of other items needed”.*

Another lowland participant noted that even if one is able to get to a LLIN distribution site during a mass distribution, bed nets may not always be available:

*“The greatest problem is that statistics taken for bed net supply doesn’t correspond to the actual nets brought for distribution hence most people don’t get the bed nets”.*

In general, after a bed net was no longer serviceable, a lowland participant indicated they would not be able to obtain another:

*“Once distributed to the people on warranty no chances of getting replacement unless you dig deep into your pockets”.*

The disparity in access was noted by a highland participant to decrease interest in obtaining a net:

“*In some region the bed nets are sold and others given freely. It therefore kills the morale of others to acquire the nets”.*

### Side effects

Unintended side effects of bed nets were noted and grouped into themes, including suffocation (H = 1, L = 3), heat (H = 1, L = 2), rash/allergy (H = 6, L = 4), family planning (H = 1), and bad dreams (H = 1). The most commonly reported theme was that exposure to the insecticide led to rash. As noted by one lowland participant:

*“Once one gets into contact with the net when the repellant is still strong, the skin rashes are developed causing discomfort to the skin”.*

‘Suffocation’ was also reported for small children. A lowland participant noted:

*“It also suffocates small children and results into difficulties in breathing”.*

When temperatures were warmer, use of bed nets was less likely as reported by a lowland participant:

*“We don’t always use a bed net because during the dry season it is hot and people do not use the bed net”.*

Interestingly, a participant from the highland site reported the belief that bed nets were a family planning tool:

*“The belief that the chemical in the bed net is for family planning [keeps them from using it]”.*

Another highland participant felt that they might cause bad dreams:

*“Sleeping under a bed net gives one to dream bad dreams”.*

### Effectiveness

The effectiveness of LLINs which had been distributed were reported as potential challenges to use and were coded into three themes: short duration of use (H = 4, L = 4), difficulty in maintaining effectiveness (H = 4), and quality of nets distributed (H = 1, L = 2). Not all brands of LLINs distributed were seen as equal in their ability to deter mosquitoes. A lowland participant noted:

*“Some kinds of the nets have less effective chemical repellent since they are not retreated and are [ineffective] even though not in bad condition”.*

Their ability to withstand regular use was questioned. A lowland participant indicated:

*“After the washing of the nets the effectiveness of the mosquito repellant fades away and that makes it not effective”.*

A highland participant noted:

*“Most nets get burned with tin lamps creating a hole making the net not effective to use.”*

### Lack of knowledge

Both highland and lowland householders commented on knowledge challenges that could influence bed net use, although highland regions reported these challenges much more commonly. Themes included lack of understanding about transmission (H = 4), lack of knowledge of how to maintain the bed net (H = 5, L = 2), and the use of LLINs for other purposes (H = 5). The value of LLINs was questioned by a highland participant:

*“[In our] culture [we] believe that we used to sleep without nets why use it now?”*

Another highland participant noted:

*“Some don’t really believe that mosquito bites from infected mosquito causes malaria leading them not to use bed net”.*

This may have been perpetuated by lack of training prior to LLIN use. A highland participant noted:

*“Those who distributed them never taught us how to use them”;* and a lowland participant indicated: “*You are just given a net at the hospital without instructions on how to use it.”*

Without proper training communities reported bed nets being used for other purposes. As a highland participant stated:

*“A bed net is a multi*-*purpose item. Some use it for decoration and curtains, fishing, rope to tie animals”.*

### Logistics

Difficulties in using a bed net were also reported, with all groups reporting difficulties in hanging (H = 6, L = 4), several on its appearance (H = 2) or ability to keep it clean (L = 2), and the perception that it should be used only with a bed (H = 2). The logistics of hanging a bed net was the most common challenge. As a lowland participant indicated:

*“Hanging a bed net is a problem especially one without a ring. It is difficult because at night when you are tired you simply sleep”.*

Using them without a bed was also reported as a potential challenge by a lowland participant:

*“Some believe that a bed net is used only on bed not on sleeping on the floor.”*

Difficulties in hanging were also noted by a lowland participant with respect to cleaning of the LLIN:

“*They become dirty very easily and they are difficult to [re]hang”.*

### Cross-sectional survey results

A total of 1923 household heads were interviewed in the highland community, Kapkangani, and 1332 household heads were interviewed in the lowland community, Miwani.

### Demographic and malaria knowledge, attitude and practice differences between sites

Demographics and attitudes towards and practices of malaria and bed nets differed between the highland and lowland sites (Table 3). In general, the lowland household heads reported having higher educated female household heads (41 vs 34 % secondary or higher), more assets (35 vs 17 % highest wealth category), and fewer children residing in the household <5 years (25 vs 53 %). In the highlands, respondents felt community members were more likely to want to buy other things (22 vs 12 %). Access to bed nets was more commonly reported as a problem in the highlands: 25 % reported it was easy to get a bed net *vs* 45 % in the lowlands and 33 vs 16 % indicated they would not be able to afford a bed net if it were not freely given. In addition, the highland respondents more commonly reported that they (9 vs 2 %) only used bed nets during the rainy season and perceived that other community members also only slept under bed nets in the rainy season (17 vs 2 %). Attitudes that there was no way to effectively prevent malaria were rare but more common in the highlands (12 vs 2 %) and respondents in the highlands were more likely to think using a bed net was difficult (13 vs 3 %).

**Table 3:** Comparison of household and individual level demographics, knowledge, and attitudes about malaria and bed nets by highland and lowland sites

Characteristic	Highland (n = 1923)	Lowland (n = 1332)
Demographics		
Children under five in household	1026 (53 %)	323 (25 %)
Pregnant woman in household	107 (11 %)	163 (12 %)
Female household head education level		
No school	211 (11 %)	170 (13 %)
Primary	1107 (58 %)	601 (45 %)
Secondary	505 (27 %)	446 (34 %)
More than secondary	90 (5 %)	115 (9 %)
Durable good ownership		
Radio	1620 (84 %)	1019 (77 %)
Stove	124 (7 %)	169 (13 %)
TV	367 (19 %)	443 (33 %)
Indoor lighting	54 (3 %)	149 (11 %)
Bike	298 (16 %)	709 (53 %)
Sofa set	795 (41 %)	626 (47 %)
Bed set	1652 (86 %)	1240 (93 %)
Wealth group		
Very low	906 (47 %)	340 (25 %)
Low	390 (20 %)	120 (9 %)
Medium	292 (15 %)	407 (31 %)
High	332 (17 %)	465 (35 %)
Basic malaria knowledge		
Malaria transmitted by mosquitoes	1910 (99 %)	1319 (99 %)
Fever a symptom of malaria	1845 (96 %)	1320 (99 %)
Malaria prevented by bed nets	1876 (98 %)	1322 (99 %)
Attitudes about malaria		
Family at risk of malaria	1871 (97 %)	1274 (96 %)
Malaria is serious	1751 (91 %)	1233 (93 %)
Children are more at risk than adults	1632 (85 %)	877 (66 %)
People in my community concerned about malaria	1881 (98 %)	1267 (95 %)
Malaria history		
Known someone who died of malaria	1025 (53 %)	777 (58 %)
Known someone with severe complications of malaria	687 (37 %)	537 (41 %)
Known someone who had long-term sequaelae from malaria	167 (9 %)	128 (10 %)
Attitude towards bed nets as a prevention strategy		
Community believes bed nets are good way to prevent malaria	1797 (94 %)	1311 (98 %)
Community rather buy other things than bed net	425 (22 %)	161 (12 %)
Community thinks bed nets purchased better than free	308 (16 %)	252 (19 %)
Householder believes bed net is beneficial	1809 (94 %)	1317 (99 %)
Bed nets are a good way to prevent malaria	1667 (87 %)	1295 (97 %)
Householder believes bed nets bought are better than free	258 (13 %)	183 (14 %)
Attitudes towards bed net access		
Same chance of obtaining a bed net	1618 (84 %)	1167 (88 %)
Bed net is easy to get	474 (25 %)	594 (45 %)
Could not afford a bed net if had to pay	632 (33 %)	219 (16 %)
Attitudes towards bed net use		
People in community only use during rainy season	335 (17 %)	28 (2 %)
Householder only uses during rainy season	171 (9 %)	29 (2 %)
No use using a net, I will get malaria anyway	235 (12 %)	32 (2 %)
Using malaria drugs is easier than using bed net	284 (15 %)	154 (12 %)
Using a bed net is difficult	241 (13 %)	36 (3 %)

### World Health Organization bed net use indicators

**Fig. 1:**
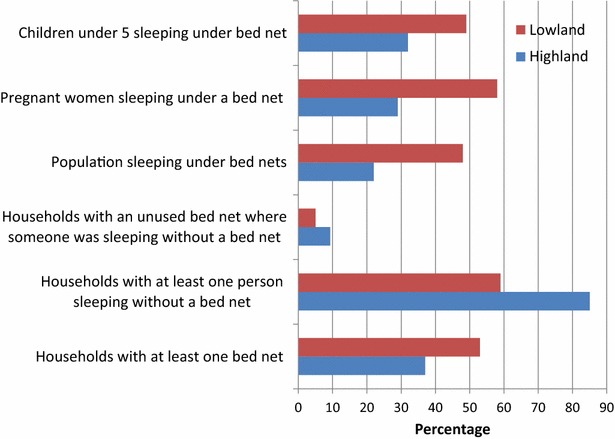
World Health Organization indicators for bed net use by community site

All World Health Organization (WHO) indicators of bed net use were lower in the highlands than in the lowlands (Fig. 1). The per cent of households owning a bed net was lower (37 vs 53 %), as was the per cent of households with at least one person sleeping without a bed net (85 vs 59 %). Lack of use of owned bed nets was also more common in the highlands than lowlands (9.3 vs 5 %). The overall proportion of the population sleeping under bed nets was 22 % in the highlands vs 48 % in the lowlands. Only 29 vs 58 % of pregnant women had slept under bed nets and 32 vs 49 % of children under five had slept under bed nets the night before. The optimal person: bed net ratio was only identified in a minority of households but was higher in the lowlands than the highlands (10 vs 5 %).

### Differences in associated ownership factors

An assessment of the factors associated with owning at least one bed net in the home indicated that there were significant differences driving ownership in the highlands compared to the lowlands (Table 4). Education was strongly correlated with owning a bed net in the highlands (OR = 3.8, p < 0.0001 for those with more than secondary school), but not in the lowlands (OR = 1.6, p = 0.10). In the highlands owning a radio was associated with a lower likelihood of owning a bed net (OR = 0.7, p = 0.05), as was owning a bed (OR = 0.4, p < 0.0001). In the lowlands owning a bed was actually positively associated with owning a bed net (OR = 1.8, p = 0.01) as was owning a bicycle (OR = 1.5, p = 0.002). In both sites having a child under age five in the household was strongly positively associated with owning a bed net [OR (H) = 1.8, p < 0.0001; OR (L) = 1.6, p = 0.0007]. Neither site had an association between bed net ownership and knowledge of malaria transmission. Perception of risk of malaria, however, was strongly associated in the highlands but not the lowlands. Respondents who perceived their family to be at risk of malaria in the highlands were 3.5 times more likely to own a bed net (p = 0.004), and the perception that malaria was a severe disease was also positively associated with owning a bed net (OR = 2.7, p < 0.0001). Respondents who felt that the community was concerned about malaria were less likely to own a bed net (OR = 0.4, p = 0.01). In the highlands, participants who knew someone who had died of malaria or suffered complications from malaria were more likely to own a bed net (OR = 1.3, p = 0.03 and OR = 1.3, p = 0.02). Respondents who reported community members would rather buy other things than a bed net were less likely to own a bed net in the lowlands (OR = 0.6, p = 0.04) and marginally associated in the highlands (OR = 0.8, p = 0.07). Household heads who believed that purchasing a bed net was better than a free one were less likely to own a bed net (OR = 0.5, p = 0.04). In the lowlands, the perception that a bed net was easy to get was associated with a higher chance of owning a bed net (OR = 1.9, p = 0.0009). In the highlands, lack of ability to afford a bed net unless it was free was associated with a lower chance of owning a bed net (OR = 0.7, p = 0.001) but in the lowlands it was associated with a higher odds of owning a bed net (OR = 1.9, p = 0.0005).

**Table 4:** Comparisons of factors associated with bed net ownership between the highland holo-endemic site and the highland seasonal transmission sites in western Kenya

	Highland seasonal transmission adjusted OR (95 % CI) (n = 1923)	Lowland holo-endemic adjusted OR (95 % CI) (n = 1332)
Demographics		
Children under five in household	*1.8 (1.5, 2.3), p < 0.0001*	*1.6 (1.2, 2.1), p = 0.0007*
Pregnant woman in household	0.8 (0.6, 1.1), p = 0.37	1.4 (1.0, 2.0), p = 0.08
Female household head education level		
No school	Ref	Ref
Primary	*1.7 (1.2, 2.5), p = 0.005*	(0.7, 1.6), p = 0.65
Secondary	*2.3 (1.5, 3.4), p < 0.0001*	1.1 (0.7, 1.6), p = 0.75
More than secondary	*3.8 (2.1, 6.9), p < 0.0001*	1.6 (0.9, 2.9), p = 0.10
Durable good ownership		
Radio	*0.7 (0.6, 1.0), p = 0.05*	1.2 (0.9, 1.7), p = 0.22
Stove	1.7 (1.0, 2.7), p = 0.06	0.8 (0.5, 1.5), p = 0.32
TV	1.2 (0.7, 2.1), p = 0.44	0.8 (0.5, 1.5), p = 0.62
Indoor lighting	1.1 (0.6, 2.1), p = 0.70	1.5 (0.9, 2.3), p = 0.09
Bike	0.9 (0.6, 1.3), p = 0.51	*1.5 (1.2, 1.9), p = 0.002*
Sofa set	0.9 (0.6, 1.5). 0.68	1.0 (0.7, 1.4), p = 0.92
Bed set	*0.4 (0.3, 0.6), p < 0.0001*	*1.8 (1.1, 3.0), p = 0.01*
Wealth group		
Very low	Ref	Ref
Low	1.4 (0.8, 2.4), p = 0.23	1.0 (0.6, 1.8), p = 0.97
Medium	1.5 (0.9, 2.6), p = 0.10	1.0 (0.6, 1.8), p = 0.96
High	2.1 (0.8, 4.8), p = 0.10	1.1 (0.5, 2.4), p = 0.79
Malaria knowledge		
Malaria transmitted by mosquitoes	1.3 (0.3, 5.4), p = 0.68	0.3 (0.04, 2.5), p = 0.27
Fever a symptom of malaria	1.4 (0.8, 2.6), p = 0.25	Undef.
Malaria prevented by bed nets	1.5 (0.6, 3.5), p = 0.34	Undef.
Malaria perception of risk		
Family at risk of malaria	*3.5 (1.5, 8.2), p = 0.004*	0.6 (0.3, 1.2), p = 0.16
Malaria is serious	*2.7 (1.7, 4.1), p < 0.0001*	0.8 (0.5, 1.4), p = 0.45
Children are more at risk than adults	1.1 (0.8, 1.5), p = 0.62	0.9 (0.6, 1.2), p = 0.35
People in my community concerned about malaria	*0.4 (0.2, 0.8), p = 0.01*	0.5 (0.3, 1.1), p = 0.09
Malaria history		
Known someone who died of malaria	*1.3 (1.0, 1.6), p = 0.03*	0.8 (0.6, 1.1), p = 0.17
Known someone with severe complications of malaria	*1.3 (1.0, 1.7), p = 0.02*	1.3 (0.9, 1.7), p = 0.15
Attitude towards bed nets as a prevention strategy		
Community believes bed nets are good way to prevent malaria	1.0 (0.6, 1.7), p = 0.90	1.3 (0.4, 2.8), p = 0.63
Community rather buy other things than bed net	0.8 (0.6, 1.0), p = 0.07	*0.6 (0.4, 1.0), p = 0.04*
Community thinks bed nets purchased better than free	1.0 (0.6, 1.9), p = 0.93	1.5 (0.9, 2.3), p = 0.09
Householder believes bed net is beneficial	1.1 (0.6, 1.9), p = 0.75	1.5 (0.3, 7.5), p = 0.67
Bed nets are a good way to prevent malaria	1.5 (0.6, 3.5), p = 0.34	0.8 (0.3, 2.2), p = 0.67
Householder believes bed nets bought are better than free	0.9 (0.7, 1.4), p = 0.54	*0.5 (0.3, 1.0), p = 0.04*
Attitudes towards bed net access		
Same chance of obtaining a bed net	0.9 (0.7, 1.6), p = 0.74	1.2 (0.8, 1.8), p = 0.33
Bed net is easy to get	1.2 (0.9, 1.7), p = 0.13	*1.8 (1.3, 2.5), p = 0.0009*
Could not afford a bed net if not free	*0.7 (0.5, 0.8), p = 0.001*	*1.9 (1.3, 2.8), p = 0.0005*
Use of other prevention methods		
House has indoor residual spray	1.1 (0.9, 1.4), p = 0.32	*1.8 (1.4, 2.3), p < 0.0001*

Italics indicate a p-value is <0.05

All results presented are from a multivariate logistic regression model stratified by site

### Factors associated with not using an available bed net

The outcome of not using a bed net in households that owned a bed net was a rare outcome. Because of this low level of reported non-use of an available bed net, analyses for examining associated factors were conducted by combining data from both study sites. Only individuals who owned a bed net were included in the analysis (n = 1408) (Table 5). After adjustment for the following potential confounders: site, children under five, pregnant women, education, and wealth, the resulting factors were associated with disuse. Owning a radio was associated with lower odds of disuse (OR = 0.5, p = 0.01). The perception that children were at higher risk than adults was also negatively associated (OR = 0.4, p < 0.001). Respondents who knew someone with severe complications of malaria were interestingly more likely to disuse a bed net (OR = 1.4, p = 0.02). Those that perceived that the general community felt bed nets were beneficial were less likely to disuse available bed nets (OR = 0.3, p = 0.008). If the respondent thought that purchased bed nets were better than freely distributed bed nets, they were more likely to report disuse of available bed nets (OR = 1.6, p = 0.05). Respondents who felt bed nets were easy to get and that they could not afford a bed net if it were not free were also more likely to disuse available bed nets (OR = 2.4, p < 0.001; OR = 1.6, p = 0.03). Disuse was also positively correlated with the perception that people in the community only use bed nets when it is the rainy season (OR = 2.8, p < 0.001, that there is no benefit to using a bed net because they will get malaria anyway (OR = 2.3, p = 0.01), that using malaria drugs is easier than using a bed net (OR = 2.2, p = 0.002), and that using a bed net is difficult (OR = 2.2, p = 0.01).

**Table 5:** Factors associated with an unused bed net being present in the house despite someone sleeping without a bed net a combined site analysis (n = 1408)

Characteristic	Crude OR (95 % CI), p = p value	Adjusted^a^ OR (95 % CI), p = p value
Demographics		
Site (lowland = reference)	*1.8 (1.2, 2.8), p = 0.004*	
Children under 5 in household	0.8 (0.5, 1.1), p = 0.17	
Pregnant woman in household	0.7 (0.3, 1.4), p = 0.29	
Female household head education level		
No school	1	
Primary	1.9 (0.7, 4.9), p = 0.18	
Secondary	2.4 (0.9, 6.3), p = 0.07	
More than secondary	1.4 (0.4, 4.5), p = 0.58	
Durable good ownership		
Radio	0.7 (0.4, 1.1), p = 0.10	*0.5 (0.3, 0.9), p = 0.01*
Stove	1.1 (0.6, 2.1), p = 0.71	0.8 (0.4, 1.6), p = 0.48
TV	*1.7 (1.1, 2.6), p = 0.009*	1.7 (0.9, 3.4), p = 0.11
Indoor lighting	0.6 (0.3, 1.5), p = 0.28	0.5 (0.2, 1.2), p = 0.11
Bike	0.8 (0.5, 1.2), p = 0.24	0.7 (0.4, 1.2), p = 0.22
Sofa set	*1.5 (1.0, 2.3), p = 0.04*	1.5 (0.8, 2.9), p = 0.26
Bed set	1.4 (0.7, 2.7), p = 0.34	1.5 (0.7, 3.1), p = 0.28
Wealth group		
Very low	1	
Low	(0.7, 2.6), p = 0.39	
Medium	1.1 (0.6, 2.0), p = 0.64	
High	1.6 (1.0, 2.7), p = 0.06	
Basic malaria knowledge		
Malaria transmitted by mosquitoes	Undefined	Undefined
Fever a symptom of malaria	0.8 (0.1, 6.1), p = 0.82	1.0 (0.1, 8.0), p = 0.98
Malaria prevented by bed nets	0.4 (0.1, 3.0), p = 0.38	2.4 (0.3, 21.8), p = 0.43
Attitudes about malaria		
Family at risk of malaria	1.7 (0.4, 7.2), p = 0.45	1.3 (0.3, 5.7), p = 0.71
Malaria is serious	0.8 (0.4, 1.9), p = 0.67	0.9 (0.4, 2.0), p = 0.78
Children are more at risk than adults	*0.4 (0.2, 0.7), p < 0.001*	*0.4 (0.2, 0.6), p < 0.001*
People in my community concerned about malaria	1.2 (0.4, 3.9), p = 0.77	0.9 (0.3, 3.0), p = 0.82
Malaria history		
Known someone who died of malaria	1.1 (0.8, 1.4), p = 0.52	0.8 (0.5, 1.2), p = 0.19
Known someone with severe complications of malaria	*1.4 (1.1, 1.9), p = 0.007*	*1.4 (1.0, 1.8), p = 0.02*
Attitude towards bed nets as a prevention strategy		
Community believes bed nets are good way to prevent malaria	*0.3 (0.1, 0.6), p = 0.002*	*0.3 (0.1, 0.7), p = 0.008*
Community rather buy other things than bed net	1.5 (0.9, 2.5), p = 0.09	1.3 (0.8, 2.2), p = 0.32
Community thinks bed nets purchased better than free	1.4 (0.9, 2.4), p = 0.13	1.4 (0.8, 2.3), p = 0.20
Household believes bed net is beneficial	0.4 (0.2, 1.1), p = 0.08	0.6 (0.2, 1.7), p = 0.35
Bed nets are a good way to prevent malaria	0.6 (0.3, 1.3), p = 0.22	0.8 (0.4, 1.8), p = 0.67
Household believes bed nets bought are better than free	*1.8 (1.1, 3.1), p = 0.02*	*1.6 (1.0, 2.5), p = 0.05*
Attitudes towards bed net access		
Same chance of obtaining a bed net	0.8 (0.4, 1.4), p = 0.41	0.8 (0.4, 1.4), p = 0.41
Bed net is easy to get	*2.1 (1.4, 3.2) 0.001*	*2.4 (1.6, 3.7), p < 0.001*
Afford a bed net if not free	*1.7 (1.1, 2.7) 0.02*	*1.6 (1.0, 2.6), p = 0.03*
Attitudes towards bed net use		
People in community only use during rainy season	*3.2 (1.9, 5.3), p < 0.001*	*2.8 (1.6, 4.8), p < 0.001*
Householder only uses during rainy season	*2.1 (1.0, 4.1), p = 0.04*	1.7 (0.8, 3.5), p = 0.16
No use using a net, I will get malaria anyway	*3.0 (1.6, 5.4), p < 0.001*	*2.3 (1.2, 4.3), p = 0.01*
Using malaria drugs is easier than using bed net	*2.5 (1.5, 4.0), p < 0.001*	*2.2 (1.3, 3.7), p = 0.002*
Using a bed net is difficult	*2.6 (1.5, 4.7), p = 0.001*	*2.2 (1.2, 4.0), p = 0.01*
Other control measures taken		
Household received indoor residual spray	0.7 (0.5, 1.1), p = 0.14	0.9 (0.6, 1.3), p = 0.59

Italics indicate a p-value is <0.05

Only households with at least one bed net present were included in the analysis. The outcome was defined as having a bed net in the household that was not being used and at least one person in the household who had not slept under a bed net the night prior

^a^Adjusted for site, education, wealth, children under five, pregnant women

### Bed net characteristics

A total of 976 bed nets in the highland seasonal transmission site were identified by the household heads and 827 in the lowlands endemic site (Table 6). Most of the bed nets had been obtained in the previous 3 years (70 % in the highlands and 90 % in the lowlands). Bed nets in the highlands were more frequently reported as being obtained from the clinic (65 %) for free or purchased from a market (26 %). The majority of bed nets were obtained in the clinics in the lowlands as well (46 %) but a larger portion were obtained during other free distributions (36 %), including mass distributions. In both sites a large majority of bed nets obtained were treated (85 % highlands and 73 % lowlands).

**Table 6:** Characteristics of bed nets owned by households in cross-sectional surveys

Bed net factor	Highlands (n = 976 bed nets) (%)	Lowlands (n = 827 bed nets) (%)
Age of bed net		
<1 year	18	39
1–2 years	52	51
3–4 years	21	4
5–10 years	8	5
10 + years	1	2
Where bed net was obtained		
Free at clinic	65	46
Free elsewhere including mass distribution	8	36
Bought at market	26	16
Bought from someone who obtained it for free	1	2
Other	0.4	0.2
Was bed net treated		
Yes	85	73
No	10	26
Unsure	5	1

## Discussion

Despite implementation of free LLIN distribution programmes, economic challenges to bed net ownership persisted in both highland seasonal transmission areas and lowland holoendemic communities in western Kenya into late 2012. While lack of using available bed nets has been a concern, it appears that, at least in the communities at the time of the study, this was relatively uncommon despite frustrations and difficulties reported by community members. Discrepancies were noted between the lowland endemic site and the highland seasonal transmission site. In general, bed net ownership was lower in the highland community and lack of using available bed nets was more frequently reported. This is not particularly surprising given that historical malaria programme strategies favoured the use of IRS in highland communities and distribution of bed nets and LLINs to lowland communities. The distribution of free LLINs in the highlands had been through the clinic and a mass distribution had not taken place until after this survey was conducted. As discussed later, this lower access to LLIN in the highland community may help explain the differences in factors associated with ownership. While the goal of the current Kenyan Malaria Strategy is to achieve universal coverage of LLINs in all geographic areas, there is still a significant gap to close [26].

Rates of LLIN ownership are highly variable across Kenya. Compared to a study conducted by Githinji et al. [27] in other areas of western Kenya, these study areas had very low ownership rates. However, the sampling strategy was different. They were recruited from clinics, while in this study the population was enumerated and a sample was taken. Individuals who report to clinics may significantly differ from the general community and likely include families with children, which was a factor associated with being more likely to own a bed net in the current study, so direct comparisons cannot be drawn.

Compared to the 2010 Kenyan Malaria Indicator Survey (KMIS) data, these data indicated a lower level of ownership in the sites under study. According to the KMIS, 70.7 % of households in the endemic regions of Nyanza Province and 59.5 % of households in highland epidemic areas of the Rift Valley Province owned at least one bed net or LLIN [21]. A similar gap was found in the present study, with the lowland site that is located in the former Nyanza Province having higher rates of bed net ownership than the highland site which is located within the Rift Valley Province. The overall lower rates may be partially explained by the difference in the household definition. In this survey individual family units residing in a separate structure were considered a household, while the KMIS defined a household as individuals who typically eat meals together. Given the structure of the family groups in the study areas, this could mean that one compound is treated as a single household in KMIS but could be divided into multiple households in the current survey. Households were defined in this manner as there is a high level of independent decision-making that occurs within an immediate family unit. Aggregation of caretakers with their extended families that reside in the same compound into a single household unit would likely lead to a higher estimate of ownership of bed nets per household.

Factors associated with bed net ownership were substantially different between the highland and lowland sites. These differences may be driven by disparities in ease of access to LLINs in the lowlands *vs* the highlands. There are other notable differences between the two sites, including maternal education, the presence of children under age five, and level of malaria transmission, the relationship between those factors and ownership were not equivalent between the two sites. In the highland site, factors associated with ownership are more reflective of the health belief model theory. The health belief model outlines five primary classes of motivating factors for health action: beliefs about the health issue, perceived benefits and barriers to action, cues to action, and self-efficacy determine a person’s health behaviour, in this case the ownership of a bed net. In the highlands, bed net owners were more likely to be educated, perceive malaria to be a serious disease that their family was at risk of contracting, have personal knowledge of someone who had suffered severe consequences of disease, and were less likely to believe that they could afford a bed net if it was not given for free. These relationships were not noted in the lowlands where ease of access was perceived to be higher: 45 vs 25 % reported it was easy to obtain an LLIN and ownership rates reflected this ease and were higher in the lowland endemic area. This could in part explain why in the lowlands there was no association between perceptions of risk and severity of malaria, or educational status. In addition multivariable models of ownership of bed nets in the highlands revealed a moderate association with wealth that was not identified in the lowland community. Other studies have demonstrated that with greater access there is a shift in factors associated with bed net ownership but have primarily focused on shifts in the wealth gap and less on associations with perceptions and attitudes about malaria [5, 8]. Further, this difference in risk factors associated with ownership does not appear to be driven by site-level differences in perceptions and attitudes towards malaria as these differences were minimal and there were no associations between these factors and ownership of bed nets (Table 2). The evidence from the cross-sectional survey is supported by the differences in barriers and attitudes reported in the community forums, with the sub-themes of access issues being more commonly reported from highland groups in addition to cultural and knowledge barriers to use.

Similar to both study sites was the association between having a child under the age of five in the household and bed net ownership. This has been demonstrated in other studies [8, 9] and is likely related to the strategy of targeting individuals at high risk for complications related to malaria, chiefly pregnant women and children under the age of five through supplemental clinic-based distributions.

Not using available bed nets was a surprisingly rare occurrence in these two communities. The definition used was restrictive as it conditioned upon: (1) the household ownership of bed nets; and, (2) having individuals that slept in the household the previous night without a bed net. Overestimates of not using available bed nets are possible without these two important restrictions. Despite its relatively rare occurrence some interesting associations were noted. Individuals who owned a radio were less likely to not use available bed nets. This may be associated with higher exposure to messaging about the importance of bed net use. Use of a radio has been associated with uptake of vector control strategies in various settings [28, 29]. The belief that the community felt bed nets were a good way to protect against malaria was highly associated with lower risk of disuse. This may reflect not only individuals’ views of bed nets but may also indicate the influence of a cultural ‘norm’; i.e., in this case, everyone is using bed nets. This is further supported by higher lack of use of available bed nets being associated with the view that community members only use their bed nets during the rainy season. Lack of using available bed nets was also positively associated with the attitude that bed nets are easy to get, bought bed nets are better than free bed nets, and that they could afford a bed net if it were not given away for free. All of these attitudes seem to centre on the theme that bed nets may have less value to those who do not use them. Past research has not found a significant difference in usage of bed nets that were purchased or received for free so this is a finding that would warrant further exploration [30]. Household heads that believed they would get malaria despite using a bed net were more likely to report not using an available bed net. An attitude that prevention is not possible e has been negatively associated with uptake of other health interventions [31, 32]. Results also imply that ease of use may play a role in whether all bed nets in a household are used. Both the attitude that malaria drugs are easier to take than using a bed net and that bed nets are difficult to use were associated with not using an available bed net. This is contrary to some studies that have indicated that while general difficulties in use may be reported, they do not influence whether a household uses a bed net [33].

## Limitations

This study is subject to several limitations. Foremost is its generalizability. This study examined two communities within western Kenya that demonstrated significant differences in their associations with bed net use as well as their knowledge, attitudes and practices in relation to malaria. This study highlights the fact that there can be significant differences between communities even within relatively close geographic proximity and demonstrates the importance of understanding the local context before conducting malaria prevention and control campaigns. Secondly, this, as in most studies related to bed net use, relied on self-report of use of existing bed nets the previous night. It is possible that individuals are aware that they should use their bed nets every night and thus report that they have used them, subsequently deflating estimates of disuse in these communities. In addition, there was no information collected on factors associated with malaria risk, which could include housing construction, use of other prevention factors (except IRS), proximity to high-risk areas, such as swamps or forest boundaries [34] which may be associated with an individual’s willingness to obtain and use a bed net. In addition, the lack of use of available bed nets analyses was limited by the rarity of the event, and this precluded using multivariable models to adjust for potentially confounding variables other than the limited set of sociodemographic variables used.

## Conclusions

Access to LLINs remained the most significant challenge facing malaria control programmes in these communities. Factors associated with ownership of bed nets differed by site emphasizing the importance of understanding local context in malaria prevention and control. To ensure equitable access to those LLINs being distributed, community health workers could deliver LLINs door-to-door instead of relying on community members to be aware of the distribution and to access the distribution sites. In these study areas, once a bed net was obtained, it was extremely likely to be used by someone in the household. However, monitoring these trends as malaria declines is important as declining use has been noted in other countries.
